# Oxygen Supplementation During Preterm Stabilization and the Relevance of the First 5 min After Birth

**DOI:** 10.3389/fped.2020.00012

**Published:** 2020-01-31

**Authors:** Inmaculada Lara-Cantón, Alvaro Solaz, Anna Parra-Llorca, Ana García-Robles, Ivan Millán, Isabel Torres-Cuevas, Maximo Vento

**Affiliations:** ^1^Neonatal Research Group, Health Research Institute La Fe, Valencia, Spain; ^2^Division of Neonatology, University and Polytechnic Hospital La Fe, Valencia, Spain

**Keywords:** preterm, fetal-to-neonatal transition, oxygen, pulse oximetry, oxidative stress

## Abstract

Fetal to neonatal transition entails cardiorespiratory, hemodynamic, and metabolic changes coinciding with the switch from placental to airborne respiration with partial pressures of oxygen of 4–5 kPa *in utero* raising to 8–9 kPa *ex utero* in few minutes. Preterm infants have immature lung and antioxidant defense system. Very preterm infants (<32 weeks' gestation) frequently require positive pressure ventilation and oxygen to establish lung aeration, a functional residual capacity, and overcome a tendency toward hypoxemia and bradycardia in the first minutes after birth. Recent studies have shown that prolonged bradycardia (heart rate <100 beats per minute) and/or hypoxemia (oxygen saturation <80%) are associated with increased mortality and/or intracranial hemorrhage. However, despite the accumulated evidence, the way in which oxygen should be supplemented in the first minutes after birth still has not yet been clearly established. The initial inspired fraction of oxygen and its adjustment within a safe arterial oxygen saturation range measured by pulse oximetry that avoids hyper-or-hypoxia is still a matter of debate. Herewith, we present a current summary aiming to assist the practical neonatologist who has to aerate the lung and establish an efficacious respiration in very preterm infants in the delivery room.

## Introduction

Fetal life elapses in a lower oxygen environment as compared to extrauterine one. Oxygen is driven from the maternal side to the fetus across the placental intervillous spaces by a positive partial pressure delta gradient of oxygen between mother and fetus. The delta gradient evolves along gestation until the third trimester when it stabilizes around 4–5 kPa (30–40 mmHg). In parallel, the lung undergoes sequential structural changes from the canalicular stage at around 24–28 weeks of gestation to the saccular stage between 28 and 34 weeks, and finally reaches the alveolar stage in the last weeks of gestation. Simultaneously, the non-enzymatic and the enzymatic antioxidant defenses and the synthesis of surfactant only reach full functionality toward the end of gestation. The lung is then prepared for *ex utero* air respiration (1). Under these circumstances, premature infants have to aerate the lung and establish a functional residual capacity and an adequate pattern of respiration. Initial breathings have to achieve a negative pressure gradient between the atmospheric and the alveolar pressures that opens the lung and extrudes the fluid filling the lung into the alveolar interstitium. The muscular weakness, the thoracic cage compliance and the lack of surfactant present in preterm infants contribute to an uneven air distribution with overdistended areas coexisting with atelectatic ones. In addition, lack of surfactant causes alveolar collapse during expiration hindering the establishment of a functional residual capacity and subsequently causing respiratory distress and respiratory insufficiency (2).

To overcome this situation, the international guidelines recommend invasive or non-invasive positive pressure ventilation using a gas admixture containing oxygen to keep the lung open and favor gas exchange (3). Despite the existing body of experimental and clinical evidence, fundamental questions such as what initial inspired fraction of oxygen (FiO_2_) is optimal, what is the optimal SpO_2_ according to the timing from birth on, and how should FiO_2_ be titrated have not been conclusively answered. Moreover, there is an increasing conviction that oxygen supplementation should be individualized according to different parameters such as antenatal complications, gestational age, type of delivery or gender (1).

The present review article summarizes the available evidence regarding the use of supplemental oxygen in preterm infants during the golden first minutes after birth aiming to assist clinicians attending newborn infants in the delivery room to achieve optimal results.

## Oxygen Monitoring and Reference Range For Oxygen Postnatal Stabilization

### Oxygen Monitoring in the Clinical Setting

Oxygen diffuses from the alveoli to the capillary blood and is distributed by the arterial vascular network bound to hemoglobin (oxyhemoglobin) or physically dissolved in blood throughout the body (4). Oxygen monitoring devices inform about the concentration of oxygen in blood and detect the degree of hypoxia or hyperoxia thus allowing the clinicians to take therapeutic decisions. Oxygen content can be assessed as partial pressure of oxygen in arterial blood samples (paO_2_). PaO_2_, probably provides the most reliable information from a physiological and pathophysiological perspective because it precisely reflects the amount of oxygen that actually reaches the tissues (5). However, the need for an arterial access and frequent blood extractions limits its applicability to monitor oxygenation in newborn infants. Transcutaneous oxygen (tcPO_2_) is also a reliable method to measure arterial blood oxygen content but has also important constraints. The electrode needs to be heated causing skin irritation and occasionally serious burns especially in extremely preterm infants. Moreover, frequent and time-consuming electrode calibration is required and readings become less precise as newborn infants get older and the skin barrier becomes thicker (6, 7). Nowadays, the most employed method to non-invasively assess arterial blood oxygen saturation (SaO_2_) is pulse oximetry (SpO_2_). SpO_2_ measures the percentage of hemoglobin (Hb) that is bound to oxygen in arterial blood. Pulse oximeters measure changes in the light absorption emitted by a transductor with two different wavelengths (red and infrared) and captured by a photodetector coinciding with pulsatile dilatation and contraction of peripheral arteries using spectrophotometric principles. Using an *ad hoc* algorithm, changes in both light transduction wave lengths are converted into arterial oxygen saturation values (8). The interpretation of the values of oxygen saturation given by the pulse oximeter correlate with the affinity of Hb for oxygen and this is clearly influenced by the amount of fetal Hb (HbF) (9). The higher the amount of HbF the higher the SpO_2_ for a given PaO_2_. In a study performed by Castillo et al. (10), oxygen saturation values between 85 and 93% correlated with a mean PaO_2_ of 56 ± 14.8 mmHg and when SpO_2_ > 93% the mean PaO_2_ was 107.3 ± 59.3 mmHg. Hence, when establishing the upper limits of SpO_2_ the risk of hyperoxemia when HbF is predominant should be taken into consideration.

Postnatal changes in SpO_2_ have been widely explored in term and preterm infants, born by vaginal or C-section, or setting up the sensor pre-or-postductal (11). SpO_2_ rises rapidly in the first 5 min to values around 80–90% and thereafter reaches a plateau around 90–95% between 6 and 10 min after birth.

### Dawson's Nomogram

**Figure 1:**
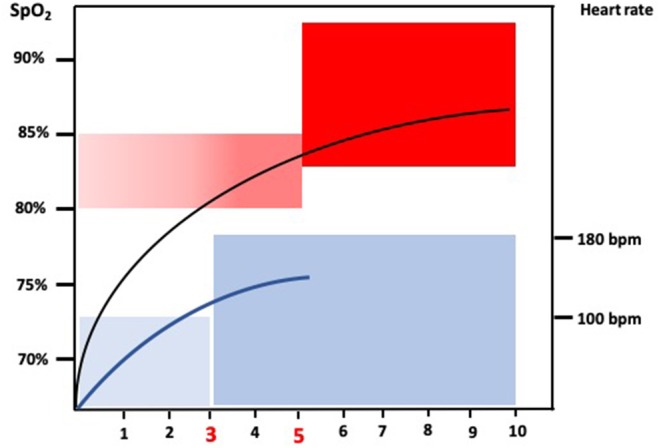
The figure depicts the recommended arterial oxygen saturation ranges based on preductal pulse oximetry measurements for newborn infants in the first 10 min after birth based on Dawson et al. (12). It also shows the recommended heart rate recommended ranges expressed in beats per minute based on Dawson et al. (18).

Dawson et al. (12) merged three different databases with a total of 468 healthy newborn infants that were continuously monitored with preductal pulse oximetry during the first 10 min after birth. None of these babies required resuscitation or oxygen supplementation. SpO_2_ interquartile ranges at 3, 5, and 10 min were 81 (71–90%), 92 (83–96%), and 97% (94–98%), respectively, for term infants and 76 (67–83%), 86 (80–92%), and 94% (91–97%), respectively, for preterm infants. However, preterm infants included were mainly late preterm infants since only few babies were below 32 weeks gestational age. Thus, no reference range for “normal” postnatal oxygenation for very preterm infants is yet available. The most recent guidelines of the American Heart Association in 2015 have recommended SpO_2_ targets at 3, 5, and 10 min of 70–75, 80–85, and 85–95%, respectively (13). AHA recommendation was based in the achievement of a preductal oxygen saturation that approximates the inter-quartile range measured in healthy term infants after vaginal delivery at sea level (80–85%) based on the study by Mariani et al. (14). Almost simultaneously, the 2015 European Resuscitation Council guidelines (15) based their targeted saturation recommendations on the 25th centile of Dawson's nomogram which represents a SpO_2_ of 85% at 5 min (12), and the Spanish Neonatal Society recommended achieving a saturation value above the 15th centile of Dawson's nomogram (75%) at 5 min (16). Although based in different reference studies and centiles, it has been widely accepted that preterm infants should achieve a SpO_2_ of 75–85% at 5 min after birth. However, preterm infants using positive pressure ventilation and air achieved targeted SpO_2_ of 80–85% significantly earlier than those breathing spontaneously which should be taken into consideration for saturation targeting (17). Figure 1 depicts the ranges for arterial oxygen saturation and heart rate (HR) measured with pulse oximetry in the first minutes after birth according to the median and ICR described by Dawson et al. (12).

## Initial Inspired Fraction of Oxygen in the Delivery Room: Contradictory Results

### Studies Supporting the Use of Higher Initial Inspired Oxygen

In 2006, the Australian/New Zealand Neonatal Society changed the policy regarding the initial inspired fraction of oxygen (FiO_2_) in the delivery room from 1.0 to 0.21 (19). Dawson et al. (20) compared postnatal adaptation of a historical cohort of preterm infants vs. a new cohort. Of note, a significant number of babies stabilized with room air needed oxygen supplementation to overcome hypoxemia in the first minutes after birth while 80% of babies resuscitated with 100% oxygen had SpO_2_ ≥ 95%. No differences in HR in the first 10 min were observed between groups (20). The Canadian Neonatal Network did a similar study comparing infants ≤27 weeks' gestation before and after 2006 when initial FiO_2_ for preterm was changed from 1.0 to <1.0 oxygen (21). Adjusted OR (AOR) for the primary outcome of severe neurologic injury or death was higher in the lower oxygen group (AOR 1.36; 95% CI 1.11–1.66) and room air (AOR 1.33; 95% CI 1.04–1.69) groups as compared with the 100% oxygen group. Besides, the randomized controlled Torpido trial compared clinical outcomes of preterm infants resuscitated with room air vs. 100% oxygen. SpO_2_ targets were 65–85% in the 5th minute after birth and 85–95% upon admission in the NICU (22). In non-prespecified analyses, preterm <28 weeks' gestation resuscitated with air had a higher mortality (RR 3.9; 1.1–13.4; *p* = 0.01); however, the investigators admitted that the study was underpowered to address this *post hoc* hypothesis reliably (22). Follow up of the Torpido trial informed of the absence of differences in death or neurodevelopmental impairment at 2 years corrected age between the group resuscitated with an initial FiO_2_ of 0.21 and 1.0 (19). Of note, in *post hoc* exploratory analyses infants who did not achieve SpO_2_ >80% at 5 min after birth were more likely to die or have neurodevelopmental impairment (OR, 1.85; 1.07–3.2; *P* = 0.03) (23).

### Studies Supporting the Use of Lower Initial Inspired Oxygen

Stola et al. (24) gradually reduced the initial FiO_2_ in the delivery room from 1.0 to 0.42, 0.28 and finally to 0.21 over a 12-month period. Compared with a historical cohort both the PaO_2_ at admission in the NICU and the median FiO_2_ employed during hospital stay were significantly lower in the study group (24). Escrig et al. (25) randomized preterm infants ≤28 weeks' gestation in the delivery room to an initial FiO_2_ of 0.3 or 0.9. Independently of the initial FiO_2_ both study groups achieved targeted saturations and HRs in the first 10 min after birth (25). However, in a subsequent study, preterm infants ≤ 28 weeks' gestation randomized to an initial FiO_2_ of 0.9 exhibited significantly higher levels of oxidative stress biomarkers and increased incidence of BPD than babies assigned to an initial FiO_2_ of 0.3 (26). Kapadia et al. (27) randomized preterm infants with gestational ages 24–34 weeks to a limited (initial FiO_2_ 0.21) vs. a high oxygen (initial FiO_2_ 1.0) strategy during DR stabilization. Both groups aimed to keep preductal SpO_2_ at 85–94%. The biochemical and clinical outcomes described by Vento et al. (26), were similar to those found in preterm infants in the high oxygen strategy group who exhibited significantly higher values for oxidative stress biomarkers and had a higher incidence of ventilator days and BPD (23). Kapadia et al. (28), recently published the follow up results of their study (27), and showed that the lower oxygen strategy was associated with improved respiratory morbidities and neurodevelopmental outcomes with no increase in mortality (28). Tataranno et al. (29) and Kato et al. (30) in blood and urine analysis, respectively, also found that using FiO_2_ of 1.0 significantly increased biomarkers of protein, lipids and DNA oxidation in preterm infants <32 weeks' gestation using high performance liquid chromatography coupled to mass spectrometry.

Two RCT studies blinded for oxygen by Aguar et al. (31) and Rook et al. (32) respectively, compared resuscitation of preterm infants with initial FiO_2_s of 0.30 vs. 0.60 and 0.30 vs. 0.65, respectively. No significant differences in achievement of targeted saturations, oxidative stress biomarkers or clinical outcomes (BPD, ROP, IVH, NEC) were reported. There was, however, an almost significant tendency toward decreased mortality in the lower oxygen group in both studies (31, 32). Follow up of these babies at 2 years of corrected age didn't show any differences in neurodevelopmental or sensory impairment among survivors (33). Oei et al. (34) published a meta-analysis comparing outcomes such as death during hospital stay or severe clinical conditions such as BPD, ROP >grade 2, IVH, ROP, PDA, and NEC of 504 preterm infants ≤28^+6^ weeks gestation who received higher (≥0.6) vs. lower (≤0.3) initial FiO_2_ in the delivery room. Mortality was lower in the masked studies of the low oxygen group (RR 0.46; 0.23–0.92; *p*=0.03) and higher in the low oxygen arms of unmasked studies (RR 1.94; 1.02–3.68; *p* = 0.04) (34). The authors concluded that there was no difference in the overall risk of death or severe morbidities depending on the initial FiO_2_ employed in extremely preterm infants. Differences in mortality between higher and lower oxygen groups were attributed to a type I error. Interestingly, babies in the low oxygen group needed more time to achieve targeted SpO_2_ (34).

### Comprehensive Updated Reviews and Meta-Analyses

Recent systematic reviews have analyzed the influence of initial FiO_2_ in preterm stabilization in the DR on death and/or relevant preterm morbidities have been published by Lui et al. (35) and Welsford et al. (36), in 2018 and 2019, respectively. The primary objective of the study by Lui et al. (35) was to determine whether lower (<0.4) or higher (>0.4) initial oxygen concentrations, when titrated according to oxygen saturation targets during postnatal stabilization of preterm infants led to improved short-and-long-term mortality and morbidity. The study followed the Cochrane Systematic Review methodology for searching and analyzing data and included randomized studies comparing lower (<0.4) vs. higher (≥0.4) initial FiO_2_ titrated to target oxygen saturations during resuscitation of preterm infants (35). The search included 914 infants the majority born before 32 weeks' gestation in 10 RCTs published between 2008 and 2016. It is relevant to note that in each of these studies SpO_2_ and heart rate were monitored and FiO_2_ titrated according to targeted saturations. No differences in mortality between both groups could be assessed (RR 1.05, 95% CI 0.68–1.63). Besides, no significant differences to relevant preterm morbidities such as ROP, PVL, IVH, NEC, BPD, or PAD could be established (35). A sub-study including two trials showed no difference in neurodevelopmental disability at 24 months corrected age between groups (35). Given the low-grade quality of the studies due to risk of bias and imprecision there is still uncertainty as to whether the use of higher or lower initial FiO_2_ affects mortality, major morbidities or long-term neurodevelopmental disability in preterm infants (35). Welsford et al. performed a systematic review and meta-analysis gathering evidence comparing different levels of FiO_2_ employed to initiate resuscitation of preterm infants who require positive pressure ventilation in the DR (36). A total of 5697 preterm infants <35 weeks' gestation participating in 10 randomized/quasi randomized controlled and 4 retrospective observational cohort studies were analyzed. Higher initial FiO_2_ was defined as ≥0.5, and lower <0.5. Results concluded that starting with either initial FiO_2_ did not influence the primary outcome of short-term mortality [risk ratio (RR) 0.83, 95% confidence interval (CI) 0.50–1.37], long-term mortality, neurodevelopmental impairment, or other morbidities (36). It should be underscored that the meta-analysis included studies from 1980 to 2018 encompassing a wide range of different guidelines, study designs, devices, and gestational ages. Hence, the use of pulse oximetry and air/oxygen blender were not routinely started until late 90's. Welsford et al. recognized relevant limitations to the review, especially due to the risk of bias and imprecision and concluded that the ideal initial FiO_2_ for preterm infants is yet unknown and requires further investigation (36).

## The Significant First 5 min After Birth

In the evaluation of postnatal adaptation of very preterm infants, HR and SpO_2_ have rendered especially valuable for predicting mortality and relevant clinical outcomes especially intraventricular hemorrhage. Virginia Apgar considered HR as the most important diagnostic and prognostic of the five signs in the Apgar score (37). Very preterm infants have physical characteristics that make clinical evaluation in the first minutes after birth difficult. Hence, although Apgar scores correlate with short-term and long-term outcomes in preterm infants there is a significant interobserver variability (38). Attempting to improve the predictability of the Apgar score gestational age and resuscitative interventions have been added to the conventional Apgar score to enhance the predictability of perinatal mortality and/or complications (39). Saugstad et al. (40) in 591 term infants resuscitated either with 21 or 100% oxygen analyzed the predictive value of different variables such as time to first breath, evolving HR, Apgar scores, SpO_2_ and base deficit. Of note, the highest odds ratio (OR) for death in the first week of life or for development of hypoxic-ischemic encephalopathy were 5 min HR ≤60 bpm (OR 16.5 for both death and HIE) and Apgar score <4 (OR 14 and 18.8) (40).

Seeking objectivity, the best means to assess HR rate from the first seconds after birth is the use of ECG. ECG is faster and more accurate that traditional stethoscope, palpation of the cord, peripheral pulses or even pulse oximeter as has been highlighted by recent systematic reviews (summarized in 40); however, existing evidence does not support substituting pulse oximetry with an ECG monitor (41). HR is the most accurate indicator of a positive response to stabilization maneuvers in the DR; hence, after a few puffs of effective ventilation and oxygenation HR rapidly increases to values above 100 bpm. Failure to do so, reveals either a profound and prolonged hypoxemia with extremely poor prognosis or an inadequate resuscitation technique or ventilatory/oxygenation parameters that need to be rapidly readjusted (42). Kapadia et al. (43) studied the association between bradycardia (HR <100 bpm) duration during preterm stabilization in the first 10 min of life with neonatal morbidity and mortality to determine if there was an interaction between prolonged bradycardia and low oxygen saturation. Results revealed that 38% of preterm infants <32 weeks' gestation experienced prolonged bradycardia and were more prone to die and/or develop IVH. Moreover, the risk of death was even higher if 5 min SpO_2_ was <80% (43). Independently of HR, the achievement of saturation targets in the first 5 min has also an important predictive value for death and/or intracranial hemorrhage. Oei et al. (44) established an association between SpO_2_ at 5 min and preterm outcomes. Individualized minute by minute data from 768 infants <32 weeks' gestation from 8 RCT of lower (<0.3) vs. higher (>0.6) initial FiO_2_ were analyzed. SpO_2_ <80% at 5 min was associated with higher risk of IVH (OR 2.04; CI 1.01–4.11; *p* < 0.05). Bradycardia at 5 min increased the risk of death (OR 4.57; CI 1.62–13.98; *p* < 0.05). It was concluded that not reaching SpO_2_ 80% at 5 min is associated with an increase rate of death or IVH (44).

The first 5 min after birth are vital for the survival of preterm infants. During this extraordinary and brief period of time, cardiocirculatory and respiratory changes to adapt to the extrauterine life are sequentially accomplished in an orderly manner. Strictly monitoring both HR and SpO_2_ are reliable means to assess if fetal to neonatal transition is adequate and if resuscitation maneuvers when necessary are being efficient. SpO_2_ readings at 5 min after birth should be incorporated to the Apgar score as an additional predictive value, and HR in the Apgar score when possible should reflect values assessed with an ECG or pulse oximeter.

## Future Studies

Delaying cord clamping (DCC) has been globally accepted as an advantageous intervention for stabilization of preterm infants in the delivery room (45). DCC increases the concentration of circulating HbF and enhances oxygen availability to tissue. Consequently, SpO_2_ ranges, targeted oxygen saturations, and oxygen titration procedure should be re-defined. Moreover, in experimental studies ventilation of the newborn while keeping the cord patent improves pulmonary circulation and oxygenation and reduces the post-asphyxia rebound hypertension which most likely protects the brain from cerebrovascular injury (46). Several clinical studies have shown the feasibility of this new approach in preterm infants; however, to date no improvement in clinical outcomes have obtained using the new modality of ventilation with intact cord when compared to the standard procedure (47–49).

The use of near infrared spectroscopy (NIRS) provides information of regional oxygen saturation and oxygen extraction of the brain almost immediately after birth and allows to better assess the impact of oxygen titration and ventilation upon cerebral oxygen metabolism. The combined use of pulse oximetry and NIRS has been successfully employed in clinical research (50, 51). Perhaps, in the future, simultaneous monitoring of brain regional oxygenation with NIRS and general oxygenation using pulse oximetry will become a standard in the delivery room.

## Conclusions

Initial FiO_2_ should avoid hyper-or-hypoxemia and overcome bradycardia in the first minutes after birth. HR and SpO_2_ should be continuously monitored in very preterm infants and readings of the monitors incorporated to the resuscitation report. In addition, we would suggest to apply interventions to overcome both low HR and SpO_2_ within 5 min after fetal expulsion which are associated with adverse outcomes.

Recent comprehensive updated reviews and meta-analyses don't show long-or-short term improvements in the use of higher or lower initial FiO_2_. However, accumulated evidence supports starting resuscitation in very preterm infants (<32 weeks' gestation) with FiO_2_ of 0.21–0.3 depending on their clinical status. Moreover, using FiO_2_ 0.3 in extremely preterm (<28 weeks' gestation) could contribute to avoid prolonged hypoxemia and bradycardia in the first five golden minutes.

## Author Contributions

All authors have contributed to the design, reference search, drafting of the manuscript, and approval of its final form.

### Conflict of Interest

The authors declare that the research was conducted in the absence of any commercial or financial relationships that could be construed as a potential conflict of interest.
